# [Retracted] Shengxian decoction decreases doxorubicin‑induced cardiac apoptosis by regulating the TREM1/NF‑κB signaling pathway

**DOI:** 10.3892/mmr.2024.13159

**Published:** 2024-01-09

**Authors:** Lei Yao, Mingtai Gui, Jianhua Li, Bo Lu, Jing Wang, Xunjie Zhou, Deyu Fu

Mol Med Rep 23: 219, 2021; DOI: 10.3892/mmr.2021.11858

Following the publication of this paper, it was drawn to the Editor's attention by a concerned reader that the majority of the Histone H3 control western blotting data featured in Figs. 2D and 4C were strikingly similar to data appearing in different form in other articles written by different authors at different research institutes.

Owing to the fact that the contentious data in the above article had already been published elsewhere prior to its submission to *Molecular Medicine Reports*, the Editor has decided that this paper should be retracted from the Journal. The authors were asked for an explanation to account for these concerns, but the Editorial Office did not receive a reply. The Editor apologizes to the readership for any inconvenience caused.

